# Waist to height ratio as a screening tool for identifying childhood obesity and associated factors

**DOI:** 10.12669/pjms.35.6.748

**Published:** 2019

**Authors:** Arda Kilinc, Nilgun Col, Beltinge Demircioglu-Kilic, Neriman Aydin, Ayse Balat, Mehmet Keskin

**Affiliations:** 1 Arda Kilinc, Department of Intensive Care Unit, Ege University, School of Medicine, Izmir, Turkey; 2 Nilgun Col, Department of Social Pediatrics, Gaziantep University, School of Medicine, Gaziantep, Turkey; 3 Beltinge Demircioglu-Kilic, Department of Pediatric Nephrology, Gaziantep University, School of Medicine, Gaziantep, Turkey; 4 Neriman Aydin, Department of Public Health, Gaziantep University, School of Medicine, Gaziantep, Turkey; 5 Ayse Balat, Department of Pediatric Nephrology, Istanbul Aydin University, School of Medicine, Istanbul, Turkey; 6 Mehmet Keskin, Department of Pediatric Endocrinology, Gaziantep University, School of Medicine, Gaziantep, Turkey

**Keywords:** Children, Obesity, Waist to height ratio

## Abstract

**Objective:**

To investigate the prevalence of obesity and associated factors during childhood in Southeastern Turkey. Another objective was to determine the cut-off points of Waist to Height Ratio (WHtR) values for defining obesity/abdominal obesity.

**Methods:**

The community-based descriptive cross-sectional study was conducted in Gaziantep Turkey between November 2011 and December 2011 with 2718 primary school/high schools students aged 6-17 years. The SPSS 22.00 was used for the analysis of data.

**Results:**

The prevalence of overweight, obesity, abdominal obesity, was 13.2%, 4.2% ,26.4%, respectively. There was a reverse relationship between BMI/WC values and sleep durations (p<0.05). The BMI/WC values were higher in students with computer usage time ≥1 hours in a day (p<0.05). Parental obesity status has an effective role on the WC/BMI values of children (p<0.05). The WHtR was a good predictor of diagnosis on obesity and abdominal obesity (AUC=0.928, p<0.0001; AUC=0.920, p<0.0001; respectively). The optimal cut-off values for obesity and abdominal obesity were detected as 0.5077, 0.4741, respectively.

**Conclusions:**

The WHtR can be used for diagnosis of obesity/abdominal obesity. Parental obesity, short sleep duration and computer use more than one hour per day are risk factors for the development of obesity in children and adolescents.

## INTRODUCTION

Childhood obesity has become one of the most serious health problems of the last century.^1^,^2^ Obesity is a multifactorial disease determined by genetic/environmental interactions (e.g. sleep duration, computer usage time, television viewing time, physical activity, and food consumption)and is an important risk factor for the development of metabolic syndrome, insulin resistance, hyperlipidemia, and cardiovascular disease.^1^-^3^ Abdominal obesity is associated with hypertension, diabetes, and dyslipidemia.^4^ Diagnosis of obesity/abdominal obesity are required age, gender, and height specific percentiles.

In recent years, waist-to-height ratio (WHtR) recommended as analternative measurement method for determining obesity/abdominal obesity in childhood.^4^ It is a simple, age-independent marker to preventing the need for age-related reference charts in different ethnic/gender groups.^5^

The aim of the present study was to investigate the prevalence of obesity, and associated factors such as parental obesity, sleep duration, television viewing/computer usage time in school children 6-17 years of age in Southeastern Turkey. Also, current study was aimed to the distribution of WHtR values, and to determine the possible cut-off points for defining obesity/abdominal obesity.

## METHODS

The community-based descriptive cross-sectional study was conducted in Gaziantep Turkey between November 2011 and December 2011 with 2718 primary school/high schools students aged 6-17 years. The schools were selected using a random cluster sampling method. The minimum sample size was determined as 2457 students at the 80% power level within α error of 5% with MedCalc (version 11.5.1) program.^6^ The study was approved by the local Ethical Committee (No: 07/2011-7, Dated: 30.06.2011) of Gaziantep University Faculty of Medicine. Healthy school children were included into the study if their parents signed the informed consent. Students with a history of chronic disease were excluded from the study.

Students completed a questionnaire which was prepared by researcher’s based on the review of the previous literature.^1^-^6^ The questionnaire form consists of two parts. The first part was related to the sociodemographic characteristics of children and their parents (such as age, sex, educational background, economic statuses and health insurance status). The second part was related to determining the factors affecting the development of obesity e.g. dietary habits, sleep pattern, duration of television/computer usage, presence of parental obesity. Parental obesity was evaluated according to BMI values, ≥25 was accepted as overweight, ≥30 was accepted as obese.^2^ The weight/height/waist circumference (WC) measurements of the students were performed using standardized protocols.

Overweight/obesity were defined according to the “International Obesity Task Force’s (IOTF)” standarts.^7^ It provides growth curves for different sex and age groups with related cut-off points. The BMI was calculated as the weight divided by square of height (kg/m²). Children were divided into groups as obese, overweight, normal weight by using age and-sex specific BMI percentile standards of Centers for Disease Control and Prevention (CDC).^8^ A BMI below the 85^th^ percentile was classified normal weight, between 85^th^ and 95^th^ percentile was classified as overweight, above the 95^th^ percentile was classified as obese. The WC ≥90^th^ percentile for children and adolescents of the same age and sex was defined as abdominal obesity according to the “International Diabetes Foundation (IDF)” diagnostic criteria.^9^ The WHtR was calculated as the WC divided by height values.

### Statistical analysis

All data were analyzed by using the computer software Statistical Package for the Social Sciences (SPSS 22.00) for Windows (Armonk, NY: IBM Corp, USA). The numeric values were given as mean ± standard deviation or percentage (%). The chi-square test was used that determine the relationship between categorical variables. Differences between groups in numeric variables were compared by Pearson correlation analysis, independent samples t-test, or bonferroni corrected ANOVA variance analysis. Receiver Operating Curve (ROC) analysis was used to assess the predictive power of WHtR for development obesity/abdominal obesity. The area under the ROC curve (AUC) ≥0.85 was accepted as correct test.^10^ P<0.05 were considered as statistically significant.

## RESULTS

The study was carried out with 2718 student (1467 male (54.0%)/1251 female (46.0%)) aged between 6.00-17.00 years (11.54±3.65 years). The 54.8% (1489) of them were adolescent age group (≥10 years’ age). The anthropometric measurements of the students and parental body mass index values were summarized in Table-I .

**Table I:** The anthropometric measurements of the students, and parental body mass index values.

	Mean±SD	Min-Max
Weight (kg)	41.10 ± 19.04	14.10-117.60
Height (cm)	143.97± 21.00	103.00-196.00
Body mass index of students (BMI)	18.62 ± 4.17	11.50-36.40
Waist circumference (cm)	66.79 ± 10.87	39.00-117.00
Waist to Height Ratio (WHtR)	0.46 ± 0.05	0.30-0.71
Maternal body mass index	27.52 ± 4.93	11.08-53.25
Paternal body mass index	26.64 ± 4.19	11.83-75.73

The prevalence of overweight/obesity/abdominal obesity was 13.2% (358)/4.2% (115)/26.4% (718). The overweight and obesity were more frequent in males (Male: overweight: 212/1467, 14.5%; obese: 70/1467, 4.8%; Female: overweight: 146/1251, 11.7%; obese: 45/1251, 3.6%; p=0.024). Abdominal obesity was more frequent in females (Female: 408/1251, 32.6%; Male: 310/1467, 21.1%; p<0.001). Overweight/obesity were more frequent in adolescents (<10 years’ age: overweight: 95/1229, 7.7%; obese: 25/1229, 2.0%; ≥10 years’ age: overweight: 263/1489, 17.7%; obese: 90/1489, 6.0%; p<0.001). Also, abdominal obesity was more common in adolescents (<10 years’ age: 265/1229, 21.6%; ≥10 years’ age: 453/1489, 30.4%; p<0.001). The BMI/WC values were lower in females(p<0.05) (Table-II). In students with abdominal obesity BMI/WHtR values were higher (p<0.05) (Table-II).

**Table II:** The assesment of the body mass index, waist circumference, waist to height ratio, sleep durations according to gender and presence of abdominal obesity.

	Mean±SD (Min-Max)	Mean±SD (Min-Max)	p

	Female	Male	
Body Mass Index (BMI)	18.31±4.04 (11.50-36.40)	18.89±4.27 (11.60-35.50)	<0.001
Waist circumference (cm)	65.70±10.42 (42.00-110.00)	67.68±11.19 (39.00-137.00)	<0.001

	*Without abdominal obesity*	*With abdominal obesity*	

Body Mass Index (BMI)	17.43±3.14 (11.50-31.60)	21.93±4.85 (11.60-36.40)	<0.001
Waist to Height Ratio (WHtR)	0.44±0.03 (0.30-0.60)	0.52±0.04 (0.44-0.71)	<0.001
Total sleep duration in weekdays (hour)	8.97±1.64 (6.00-12.00)	8.55±1.54 (6.00-10.00)	<0.001
Total sleep duration in weekend days (hour)	9.82±1.70 (7.00-14.00)	9.58±1.85 (7.00-12.00)	0.013

There was a reverse relationship between BMI/WC values and sleep durations of students in week days (r=-0.357, p<0.001; r=-0.372, p<0.001; respectively). Similar association was observed between sleep durations in weekend days (r=-0.213, p<0.001; r=-0.222, p<0.001; respectively). In students with abdominal obesity sleep durations were lower (p<0.05) (Table-II). The WC/BMI values were higher in students with computer usage time ≥1 hours in week days’/weekend days (Table-III). Snack consumption between meals was a risk factor on BMI and WC values (p<0.001) (Table-IV). Similarly, regularly breakfast was also a risk factor on BMI/WC/total body fat (kg) values (p<0.001) (Table-IV).

**Table III:** The relationship between screen time and body mass index, waist circumference, waist to height ratio values.

	<1 hour Mean±SD (Min-Max)	≥1 hour Mean±SD (Min-Max)	p
***Computer usage time***			
***In weekdays***			
Body Mass Index (BMI)	18.83±4.09 (11.70-35.00)	20.37±4.32 (12.40-36.40)	<0.001
Waist circumference (cm)	67.37±10.43 (47.00-109.00)	70.65±11.41 (50.00-107.00)	0.001
***In weekend days***			
Body mass index (BMI)	18.79±3.96 (12.00-34.40)	19.68±4.25 (11.70-36.40)	0.001
Waist circumference (cm)	66.69±9.82 (47.00-105.00)	69.66±10.98 (47.00-110.00)	0.001
***Television viewing time in weekdays***			
Waist to Height Ratio (WHtR)	0.4613±0.4907 (0.36-0.69)	0.4670±0.5344 (0.30-0.71)	0.045

**Table IV:** The relationship between regularly breakfast/snack consumption and body mass index, waist circumference, total body fat values.

	Yes [Mean±SD (Min-max)]	No [Mean±SD (Min-max)]	p
***Regularly breakfast***			
Waist circumference (cm)	64.71±10.25 (42-109)	69.39±11.1 (49-110)	<0.001
Body mass index (BMI)	17.90±3.90 (11.70-36.40)	19.79±4.53 (12.80-35.00)	<0.001
Total body fat (kg)	6.73±5.69 (0.50-41)	9.31±7.18 (0.60-38)	<0.001
***Snack consumption***			
Waist circumference (cm)	64.89±10.16 (42-110)	68.16±10.73 (50-109)	<0.001
Body mass index (BMI)	18.00±3.98 (11.70-36.40)	19.17±4.04 (11.60-31.00)	<0.001

The effect of the parental obesity status on anthropometric measurements of the students were evaluated. Each of the parents’ obesity status independently has an effective role on the WC/BMI values of the children. The BMI/WC values were higher in students with obese mothers/fathers (p<0.05) (Table-V). When obesity status of both parents was evaluated with together, differences between the three groups was statistically significant in the BMI and WC. Only maternal obesity was effective factor on the WHtR (Table-V).

**Table V:** The association between parental obesity status and waist circumference, body mass index, waist to height ratio values of the students.

	Mean±SD (Min-Max)	Mean±SD (Min-Max)	Mean±SD (Min-Max)	p
***Maternal weight classification***				
	***Normoweight***	***Overweight***	***Obese***	
Body Mass Index (BMI)	16.91±3.29* (11.60-31.00)	18.58±3.96^†^ (11.70-34.30)	19.69±4.55^‡^ (12.10-35.00)	<0.001
Waist circumference (cm)	62.66±9.09* (43.00-109.00)	66.43±10.22^†^ (44.00-107.00)	68.74±11.07^‡^ (50.00-110.00)	<0.001
Waist to Height Ratio (WHtR)	0.46±0.04 (0.32-0.69)	0.46±0.04^†^ (0.36-0.70)	0.47±0.53^‡^ (0.35-0.71)	0.004
***Paternal weight classification***				
	***Normoweight***	***Overweight***	***Obese***	
Body Mass Index (BMI)	17.29±3.59* (11.70-34.80)	18.47±3.90^†^ (11.60-34.40)	19.73±4.64^‡^ (11.70-35.00)	<0.001
Waist circumference (cm)	63.75±9.63* (42.00-110.00)	66.27±10.25^†^ (43.00-105.00)	68.31±10.96^‡^ (44.00-109.00)	<0.001
Waist to Height Ratio (WHtR)	0.46±0.04 (0.35-0.71)	0.46±0.05 (0.32-0.68)	0.47±0.55 (0.36-0.70)	>0.05
***Weight classification of both parents (Obese= overweight +obese)***				
	***Both parents are normoweight***	***One of the parent is obese***	***Both parents are obese***	
Body Mass Index (BMI)	16.31±2.93|| (11.70-27.60)	18.03±3.83§ (11.60-34.80)	19.38±4.33¶ (11.70-35.00)	<0.001
Waist circumference (cm)	61.52±8.92|| 849.00-98.00)	65.22±9.91§ (43.00-110.00)	68.06±10.78¶ (44.00-105.00)	<0.001
Waist to Height Ratio (WHtR)	0.46±0.04 (0.35-0.60)	0.46±0.05 (0.322-0.71)	0.46±0.05 (0.35-0.70)	>0.05

* Significant overweight versus normoweight,

† Significant obese versus overweight,

‡ Significant obese versus normoweight, || Significant one of the parent is obese versus both parents are normoweight, § Significant both parents are obese versus one of the parent is obese, ¶ Significant both parents are obese versus both parents are normoweight.

**Fig. 1:**
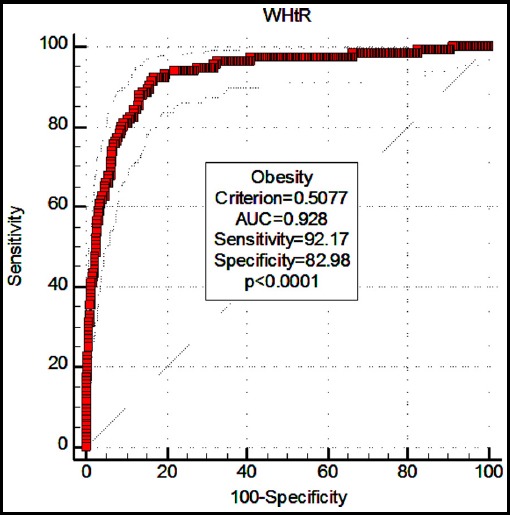
The ROC curve analysis for the role of WHtR in diagnosing obesity.

**Fig. 2:**
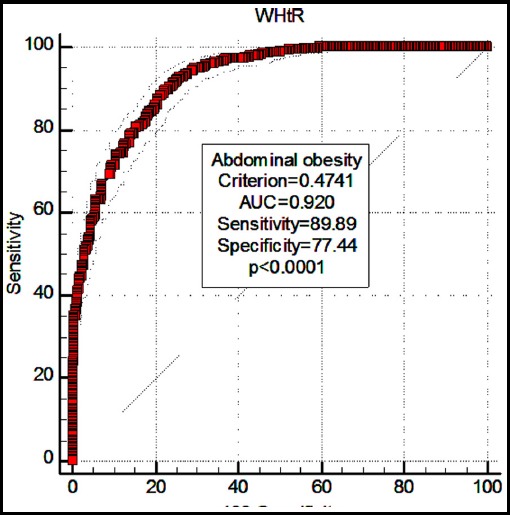
The ROC curve analysis for the role of WHtR in diagnosing abdominal obesity.

The ROC curve analysis indicated that WHtR was a good predictor of diagnosis on obesity/abdominal obesity (AUC=0.928, p<0.0001; AUC=0.920, p<0.0001; respectively). The optimal cut-off values for obesity/abdominal obesity were detected as 0.5077, 0.4741, respectively (Fig. 1, Fig. 2). Table-VI presents the optimal cut-off values of WHtR for identifying overweight, obesity, and abdominal obesity according to age/gender.

**Table VI:** Optimal cut-off points of waist to height ratio for identifying abdominal obesity, overweight, and obesity in childhood.

Abdominal Obesity					

Waist to Height Ratio (WHtR)	All students (n=2718)	Girls (n=1251)	Boys (n=1467)	Children (n=1229)	Adolescent (n=1489)
Cut-off points	0.4741	0.4697	0.4762	0.4959	0.4645
Area under Curve (AUC)	0.920	0.907	0.940	0.926	0.964
Sensitivity	89.89	90.05	92.58	89.51	93.19
Specificity	77.44	74.76	78.97	82.61	87.93
P	<0.0001	<0.0001	<0.0001	<0.0001	<0.0001

*Overweight/Obesity*					

*Waist to Height ratio (WHtR)*	*All students* *(n=2718)*	*Girls* *(n=1251)*	*Boys* *(n=1467)*	*Children* *(n=1229)*	*Adolescent* *(n=1489)*

Cut-off points	0.4769/ 0.5077	0.4766/ 0.5378	0.4767/ 0.5077	0.4922/ 0.5133	0.4693/ 0.5084
Area under Curve (AUC)	0.819/0.928	0.833/0.907	0.816/0.942	0.800/0.930	0.882/0.940
Sensitivity	79.49/92.17	86.39/80.00	76.60/95.71	78.83/92.00	80.17/91.11
Specificity	70.47/82.98	64.91/93.37	73.50/83.82	69.88/81.23	81.16/88.71
P	<0.0001/ <0.0001	<0.0001/ <0.0001	<0.0001/ <0.0001	<0.0001/ <0.0001	<0.0001/ <0.0001

## DISCUSSION

Increasing prevalence of obesity is an important health issue in childhood.^1^,^2^ Our study demonstrated that the prevalence of overweight/obesity in 6-17 years’ school children were 13.2%/4.2%. Another studies of different region from Turkey reported that, overweight/obesity prevalence was 9.42%-13.9%/8.0%-13.9% in 7-15 years old children.^11^,^12^ This difference may be associated with local eating style and restricted physical activity. In various studies from different countries have been reported that, 13.1-13.9% of the children were classified overweight, 5.0%-10.7% were classified obese.^3^,^13^-^15^ It is not easy to compare the results of previous studies because of differences in age range, variety of study populations, sample size, ethnicity, and different definitions of obesity.^1^-^3^ Also, specific ethnic groups have an obesity tendency in childhood. Ethnic differences in overweight/obese preschool children seem to have their origins that prenatal (maternal educational level), natal (parental BMI), and postnatal (baby’s weight gain) factors. Postnatal BMI development is very similar among ethnic groups.^2^

In recent years, abdominal obesity has been accepted as more accurate predictor of obesity related co-morbidities.^4^ We demonstrated that, 26.4% of the students had abdominal obesity. The prevalence of abdominal obesity is reported 11.1% to 25.9% in different studies in childhood/adolescence period.^13^,^15^-^18^ It is very difficult to compare WC values in different studies, because of different definitions, and WC values may be measured at different body sites and there is no consensus on which region is optimal.

The influence of gender on the BMI is controversial.^11^ Inanc et al. reported that obesity prevalence was higher in girls aged 7-15 years.^12^ Some studies, did not show any significant association between sex and BMI values.^19^,^20^ On the other hand, the prevalence of overweight/obesity were more frequent in boys aged 7-17 years.^11^,^21^ It is not clear the potential reasons the differences between BMI and gender. Pubertal period is a critical period for body fat distribution. Adipose tissue is disseminated in upper body part in male while in lower body part in female, and girls tend to carry more body fat than boys at the same BMI.^2^,^17^ Waist circumference and BMI values tend to increase with age among both gender.^11^,^14^,^16^,^18^ In accordance with the literature, we found that obesity/abdominal obesity were more common in adolescents.

The BMI may be inadequate to distinguish between excess body fat or high muscle mass.^1^,^4^ Abdominal adiposity indicated by WC is found to be more associated with health risks such as metabolic (dyslipidemia and insulin resistance) and cardiovascular complications than general adiposity. The WC is considered as a practical/cheap anthropometric measurement, giving relevant information about body fat distribution and reflecting the degree of central obesity.^1^ If WC values are corrected with the height of person, determining power for obesity-related cardiovascular/metabolic risk factors improves.^4^ Bacopoulou et al. reported that WHtR was a good predictor of general/central adiposity.^17^ Also, WHtR offers advantages of same boundary values which can be used for both gender/age in different ethnic groups.^4^,^5^,^21^ It has been suggested that the same cut-off value of 0.5 may be used in all age groups in children and adolescents since WHtR is only poorly associated with age.^5^ Optimal cut-off values of WHtR for obesity/abdominal obesity were detected as 0.50/0.47 in our study. Similarly our results, different studies from different countries showed that WHtR (simple, easy, and accurate index) higher than 0.5 has been proposed as a cut-off point for diagnosis obesity/abdominal obesity in both gender and at all ages.^5^,^17^,^18^,^22^ When compared to other studies, the sensitivity and specificity of our WHtR values was similar to that seen in Greek high school adolescents.^17^ Mushtaq et al. demonstrated that WHtR cut-off ≥0.5 for defining central obesity corresponded to 85^th^ WHtR percentile irrespective of age and gender in Pakistani children.^18^ So, it may be recommended as an alternative measure in the assessment of obesity/abdominal obesity, especially in pediatric primary care practice, because it may eliminate the need for age-related reference charts. It is also suitable as a screening tool for population and epidemiologic studies as it is a relatively age-independent measurement method.^15^ In addition, Mishra et al. has demonstrated in India that high WHtR (≥0.5) is associated with high blood pressure in school children.^15^ Zhang et al. has reported from China, the prevalence of high blood pressure increases with WHtR values in children and adolescents.^16^ Meseri et al. showed that 0.55 was the optimal cut-off point of WHtR for cardiovascular disease risk in Turkish adults for both gender.^22^ We believe that this is the first study examining the WHtR values among children and adolescents in a large population in Southeastern Turkey, but further studies in different regions of Turkey are needed.

Consistent with our results, short sleep duration was found to be associated with an increased risk of childhood obesity.^2^,^23^,^24^ This relationship may be due to changes in the sympathetic nervous system, circadian rhythm, and hormone concentrations. Neurohormonal changes in periods of reduced sleep may affect appetite.^3^,^23^ Short sleep duration also allows more time to consume foods, reduces daytime physical activity as it causes fatigue, and increases computer usage/television viewing time (screen time).^3^,^23^,^24^ Long periods of screen time were associated with childhood obesity.^2^,^21^ The American Academy of Pediatrics recommends that limit the screen time max two hours in a day as a preventive measure for health problems.^25^ But our study draws attention that BMI/WC values were higher in students with computer usage time ≥1 hours in a day. Further researches are needed to confirm our findings.

The main strength of this study is that it has provides additional data about optimal thresholds of WHtR for diagnosis of obesity/abdominal obesity. Another strength is that; anthropometric measurements are performed using standardized protocols from a large sample. Additionally, examines the relationship between familial obesity/sleep durations/screen time and obesity risk in childhood.

### Limitations of the study

First; pubertal status of the students is not evaluated. Another limitations relates to use of self-reports sleep and screen time habits of students and parental height/weight status. Therefore, these reports may not be objective.

## CONCLUSION

Our results provide an easy, simple, accurate and age-independent marker to preventing the need for age-related reference charts in different ethnic/gender groups. The optimal cut-off values of WHtR for obesity/abdominal obesity are detected as nearly 0.50 in both gender, children, and adolescents. These findings also emphasize the importance of the parental obesity, short sleep duration and computer use more than one hour per day on the development of obesity in children and adolescents.

### Author`s contribution:

**AK** performed anthropometric measurements.

**NC** conceptualized/designed the study, drafted the manuscript and takes responsibility for integrity of research.

**BDK, NA, AB and MK** revised the manuscript critically.
